# World and European Rowing Medallists Pace With Smaller Variation Than Their Competitors

**DOI:** 10.3389/fspor.2021.790198

**Published:** 2021-12-22

**Authors:** Fredrik Mentzoni, Thomas Losnegard

**Affiliations:** ^1^The Norwegian Olympic Sports Center (Olympiatoppen), The Norwegian Olympic and Paralympic Committee and Confederation of Sports, Oslo, Norway; ^2^Department of Physical Performance, Norwegian School of Sports Sciences, Oslo, Norway

**Keywords:** pacing, rowing, strategy, race analysis, endurance, performance level, split times, hydrodynamics

## Abstract

**Purpose:** To establish the relation between pacing pattern and performance, within sex, and number of crew members, at the very highest performance level in World class rowing.

**Methods:** Pacing profiles based on official 500 m split times in 106 A-finals with six contesting boat crews (*n* = 636 crews), in recent World (2017–2019) and European (2017–2021) championships, were analyzed. The coefficient of variation (CV) and sum of relative differences (SRD) of the split times, and normalized velocities in the four segments of the race, were compared between performance levels, that is, placement (1st–6th), and subgroups based on sex (female or male) and number of crew members (one, two, or four). Statistical tests and resulting *p*-values and effect sizes (Cohen's *d*) were used to assess differences between groups.

**Results:** The pacing profiles of the medallists had smaller variation than those of the non-podium finishers (CV = 1.72% vs. CV = 2.00%; *p* = 4 × 10^−7^, *d* = 0.41). Compared to the non-podium finishers, the medallists had lower normalized velocities in the first and second segments of the race, slightly higher in the third segment and higher in the fourth segment. Female crews paced somewhat more evenly than male crews. No significant differences were found in the evenness of pacing profiles between singles, doubles/pairs and quads/fours. Analyses of SRD were overall consistent with analyses of CV.

**Conclusion:** Medal winners in major rowing championships use a more even pacing strategy than their final competitors, which could imply that such a strategy is advantageous in rowing.

## Introduction

An athlete's pacing pattern is widely recognized to have a substantial influence on the performance in endurance sports (Abbiss and Laursen, 2008; Tucker, 2009; Roelands et al., 2015; Casado et al., 2021). As the pacing pattern composed by the athlete directly influences the energy turnover rate and thereby performance, a variety of patterns (e.g., negative, all-out, positive, parabolic, and variable) exists. In endurance sports of short durations (≲2 min), all-out and positive pacing profiles are advantageous and common, e.g., ≤800 m running (Tucker et al., 2006) and ≤ 200 m swimming (Menting et al., 2019). It has been suggested that for events lasting longer than 2 min, an even pacing strategy may be optimal to achieve the best time or highest mean power output (Abbiss and Laursen, 2008). In practice, this seems evident at least for relatively long durations (≳ 10 min), e.g., ≥5,000 m running (Tucker et al., 2006; Diaz et al., 2018). For events lasting approximately 2–10 min, the literature and applied practice seem less certain. Typically, various parabolic (U-, J-, or reverse J-shaped) pacing profiles are observed (Menting et al., 2019; Casado et al., 2021). Interestingly, several best times and world records are set with small variations in pace and relatively even profiles, e.g., 5,000 m speed skating (≈6 min) (ISU, 2020) and 4,000 m track cycling (≈4 min) (Tokyo Olympics, 2021). Moreover, in various sports, the more “calculated” even pacing pattern has occurred over the recent years, making the even pacing more even, cf. e.g., Foster et al. (2014) and Diaz et al. (2018). However, as stated by Casado et al. (2021), pacing profiles within sports differ as well as the type of competition (championship; finals vs. qualifications, goal to set best times) implying the need for study of pacing profiles in the sport specific content.

The finishing time in rowing is always more than at least 5 min in 2000 m events, that is, World Rowing's standard regatta distance, which is used in all major championships. The importance of viscous fluid (hydro- and aerodynamic) drag yields a highly non-linear relation between mechanical power output and velocity. Consequently, the energetic cost of an uneven pacing strategy may be higher than in sports where the total resistive force is less dominated by viscous drag, e.g., running (gravity), cross-country skiing (gravity and snow-ski-friction) or uphill cycling (gravity) in sufficiently steep slopes (van Druenen and Blocken, 2021). Therefore, an even pacing strategy seems the most obvious choice to optimize performance in rowing. In contrast, previous studies, that have used 500 m split times to assess various aspects of pacing strategies in rowing, agree that, typically, a reverse J-shaped pacing profile (fastest-slow-slow-fast) is applied in 2,000 m rowing (Garland, 2005; Brown et al., 2010; Muehlbauer et al., 2010; Muehlbauer and Melges, 2011). Interestingly, a fast-start approach is also found in long-distance rowing (Edwards et al., 2016).

However, it is unclear if rowers at the highest performance level will seek toward more even pacing strategies (flatten their reverse J), to further increase performance. The study by Brown et al. (2010) supports the hypothesis that there is a relation between performance level and pacing strategy. In contrast, Garland (2005) did not find any difference in pacing profiles between winners and the rest of the heat. Furthermore, the study by Muehlbauer and Melges (2011) confirmed that there are significant differences in pacing patterns in heats and finals. Thompson (2014) summarized that previous studies of pacing in rowing have found similar pacing profiles independent of finishing position. Notably, previous studies have proved differences in pacing profiles between performance levels in some sports, e.g., running (Hettinga et al., 2019), and insignificant differences in pacing between performance levels in other sports, e.g., 1,500 m swimming (Lara and Del Coso, 2021). Further, Losnegard et al. (2016) found that the pacing pattern depended on performance in cross-country skiing among males, but not among females.

Inspired by the previous studies of split times, we analyze pacing profiles, from recent World (2017–2019) and European (2017–2021) championships, in the present study. Official 2,000 m race times, as well as intermediate split times at each 500 m mark, are obtained from the website of the World Rowing Federation. In our study, we limit the analysis to A-finals only, in order to investigate the split time characteristics and pacing profiles at the very highest performance level and to limit tactical behaviors often seen in the qualifications. This obviously limits the number of events and boat crews, which we compensate for by assessing eight recent championships.

Our main objective was to establish the relation between rowing performance, in terms of the final placement, and the pacing strategy. Furthermore, we tried to establish whether or not the pacing strategy in the considered A-finals is a function of sex and/or the number of crew members in the boat class.

## Materials and Methods

### Dataset and Inclusion Criteria

The analysis included some of the most contested boat classes in the World championships of 2017, 2018, and 2019 (W17–W19) and the European championships of 2017, 2018, 2019, 2020, and 2021 (E17–E21). The considered boat classes were the lightweight single sculls (LM1x, LW1x), the lightweight double sculls (LM2x, LW2x), the single sculls (M1x, W1x), the coxless pairs (M2-, W2-), the double sculls (M2x, W2x), the coxless fours (M4-, W4-), and the quadruple sculls (M4x, W4x). This includes all Olympic rowing classes, except M8+/W8+ which we did not include in the analysis due to several finals with less than six contesting crews.

Four criteria were applied to the dataset. (1) Only A-finals were considered to ensure that the contesting crews were at the highest performance level, that the athletes were highly motivated to go as fast as possible for the whole race distance, and that the winners were indeed World or European champions. (2) All 500 m segment times needed to be available on the website of the World Rowing Federation. (3) Only A-finals with six contesting boats were included. (4) Only A-finals with a difference in time between the 1st and 6th place smaller than 30 s were considered. Criteria (3) and (4) were applied to ensure comparable conditions between the different events and to avoid extreme outliers. In the rowing classes and events considered, a total of 106 finals (636 boats) fulfilled the criteria. A list of the rowing classes and events included in the analysis is provided in Table 1.

**Table 1:** Included A-finals; events and boat classes.

	**E17**	**E18**	**E19**	**E20**	**E21**	**W17**	**W18**	**W19**
LM1X	-	-	-	-	-	-	-	-
LM2X	-	-	-	-	-	-	-	-
LW1X	-	3	-	-	-	-	4	-
LW2X	-	-	-	-	-	-	-	-
M1X	-	-	-	-	-	-	-	-
M2-	-	4	-	-	-	-	-	-
M2X	-	-	-	2	-	-	-	-
M4-	-	-	-	-	-	-	-	-
M4X	-	-	-	-	-	-	-	-
W1X	-	-	-	-	-	-	-	-
W2-	-	-	-	-	-	4	-	-
W2X	-	-	-	-	-	-	-	-
W4-	3	-	-	-	-	-	-	-
W4X	-	-	-	-	-	-	-	-

*Of the 112 possible A-finals, six are excluded for the following reasons (cf. criteria in text): 2) Not all 500 m split times were available; 3) Less than six boats in the final; 4) More than 30 s between 1st and 6th place. A cell with a - indicates that the A-final was included in the dataset*.

### Pacing Profile Assessment

To assess the pacing profiles, analyses were made based on the time spent on each of the four 500 m segments. In the following, a segment time is denoted *t*_*j*_; *j* = 1, ..., 4, where *j* = 1 is the first segment from 0 to 500 m, *j* = 2 is the second segment 500–1,000 m and so forth. Further, $\bar{t}$ is the arithmetic mean of the segment times. The pacing pattern was assessed through the standard deviation of the segment times, i.e., the square root of the average of the squared deviations from the mean segment time,


(1) $$\begin{array}{l} t_{std}=\sqrt{\frac{1}{4}\overset{n\;=\;4}{\underset{j\;=\;1}{\sum }}{\left(t_{j}-\bar{t}\right)}^{2}}. \end{array}$$

The coefficient of variation (CV) allows for comparisons of the relative variation for different mean segment times, hence, different race times,


(2) $$\begin{array}{l} CV=\frac{t_{std}}{\bar{t}}. \end{array}$$

Consequently, if CV is large, the standard deviation of the segment times is relatively large compared to the mean of the segment times, indicating that the rower's average speed in the four segments varied relatively more than if CV is small. Note the importance of squaring of terms in Equation (1); if one split time is more different to the mean than the other split times, this split time can dominate the standard deviation (and CV) considerably. As an alternative to CV, we also analyzed the sum of the absolute differences of the split times and the mean split time, relative to the mean split time, here denoted SRD (sum of relative differences),


(3) $$\begin{array}{l} SRD=\frac{\sum_{j\;=\;1}^{n\;=\;4}|t_{j}-\bar{t}|}{\bar{t}}. \end{array}$$

Furthermore, the normalized velocity for each 500 m segment, *v*_*j*_, yields a comparison between the mean velocity of a 500 m segment, *u*_*j*_, and the mean velocity of the full 2,000 m race distance, $\bar{u}$, which is equivalent to a comparison of the mean segment time, $\bar{t}$, and the time spent on a given segment, *t*_*j*_,


(4) $$\begin{array}{l} v_{j}=\frac{u_{j}}{\bar{u}}=\frac{\frac{500m}{t_{j}}}{\frac{2000m}{\sum_{j\;=\;1}^{n\;=\;4}t_{j}}}=\frac{\frac{1}{4}\sum_{j\;=\;1}^{n\;=\;4}t_{j}}{t_{j}}=\frac{\bar{t}}{t_{j}}. \end{array}$$

The normalized velocity yields a comparison of a crew's mean velocity in each 500 m segment compared to the same crew's mean velocity for the full 2,000 m race distance. Moreover, we use the relative race time, *t*_*rel*_, of a crew to express the race time of that crew compared to the mean race time of all six crews in the final. This quantity may also be expressed in terms of the mean split times, as is done in the following,


(5) $$\begin{array}{l} t_{rel}=\frac{\bar{t}}{\frac{1}{6}\sum_{k\;=\;1}^{m\;=\;6}{\bar{t}}_{k}}, \end{array}$$

with ${\bar{t}}_{k}$; *k* = 1, ..., 6, being the mean split times of the six crews in the final.

### Statistical Analysis

Analyses and calculations were performed in Python (version 3.8.3). The statistical tests applied functions of the stats package of SciPy (version 1.7.0) and Pingouin (version 0.5.0). A *p* ≤ 0.05 was considered statistically significant. Welch's *t*-test for the means of two populations (scipy.stats.ttest_ind) was used to compare two groups, e.g., CV of female crews vs. male crews. Comparisons were made with analysis of variance (ANOVA) methods using the statsmodels and Pingouin packages. The chi-square test of independence of variables in a contingency table (scipy.stats.chi2_contingency) was used to test the independence of the distribution of pacing profile types between different subgroups of the dataset (e.g., distribution among female crews vs. distribution among male crews).

## Results

### Pacing Profile Characteristics

**Figure 1:**
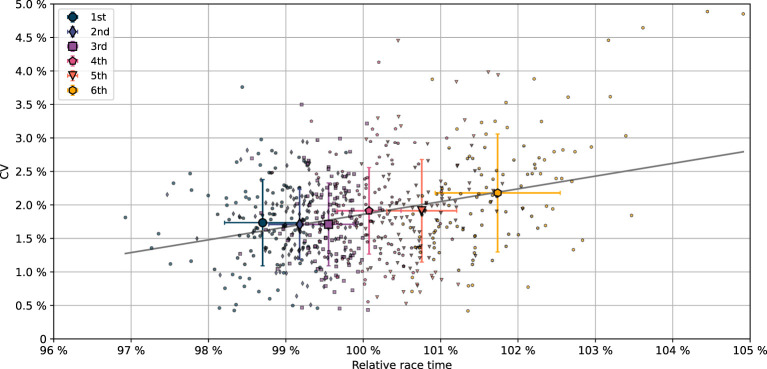
The coefficient of variation (CV) of the split times as function of the relative race time, i.e., the race time of a crew compared to the mean race time in that final, c.f. Equation (5). The results include 636 boat crews from 106 A-finals. Different colors and markers are used for the six finishing placements; mean values per placement are presented with bigger markers; error bars indicate one standard deviation, cf. legend. Linear regression analysis yields intercept equal to −0.172 and slope equal to 0.190 (gray solid line); *r* = 0.30, *p* = 5 × 10^−15^, i.e., the slope of CV against relative race time is non-zero with *p* = 5 × 10^−15^.

The coefficient of variation (CV) of the segment times is presented in Figure 1 as a function of the relative race time, i.e., the race time of a crew compared to the mean race time in that final, cf. Equation (5). The CV (mean and standard deviation) per placement is provided in Table 2. Each group of medallists (1st, 2nd, 3rd place) had smaller mean CV than each group of non-podium finishers (4th, 5th, 6th place). The difference in mean CV (standard deviation) between the medallists, CV = 1.72% (0.60%), and the non-podium finishers, CV = 2.00% (0.78%), was significant (*p*= 4 × 10^−7^, *d* = 0.41), indicating less variation in the time spent on each of the four 500 m segments among the medallists compared to the non-podium finishers. The sum of the relative differences (SRD), Equation (3), is included in Table 1. Consistent results are found between CV and SRD; linear regression yielded *r* = 0.98 based on the *n* = 636 crews. Each group of medallists had smaller mean SRD than each group of non-podium finishers. The difference in mean SRD (standard deviation) between the medallists, SRD = 6.0% (2.1%), and the non-podium finishers, SRD = 6.9% (2.6%), was significant (*p*= 4 × 10^−6^, *d* = 0.37).

**Table 2:** Mean CV (standard deviation), mean SRD (standard deviation), Equation (3), and the mean standard deviation, *t*_*std*_, of the four segment times, and mean normalized velocities in the four segments of the race, 0–500 m (*v*_1_), 500–1,000 m (*v*_2_), 1,000–1,500 m (*v*_3_), and 1,500–2,000 m (*v*_4_), as function of performance (final placement).

	**CV [%]**	**SRD [%]**	***t*_*std*_**	***v*_1_ [%]**	***v*_2_ [%]**	***v*_3_ [%]**	***v*_4_ [%]**
1st	1.73 (0.64)	6.0 (2.3)	1.73 s	102.3	99.0	98.7	100.2
2nd	1.71 (0.52)	6.1 (1.9)	1.71 s	102.1	98.8	98.6	100.7
3rd	1.71 (0.62)	6.0 (2.1)	1.72 s	102.0	98.8	98.6	100.6
4th	1.91 (0.65)	6.7 (2.3)	1.94 s	102.5	98.9	98.4	100.4
5th	1.91 (0.77)	6.6 (2.6)	1.95 s	102.7	99.2	98.6	99.7
6th	2.18 (0.88)	7.4 (3.0)	2.25 s	103.3	99.5	98.5	98.9

*Results based on 106 A-finals*.

**Figure 2:**
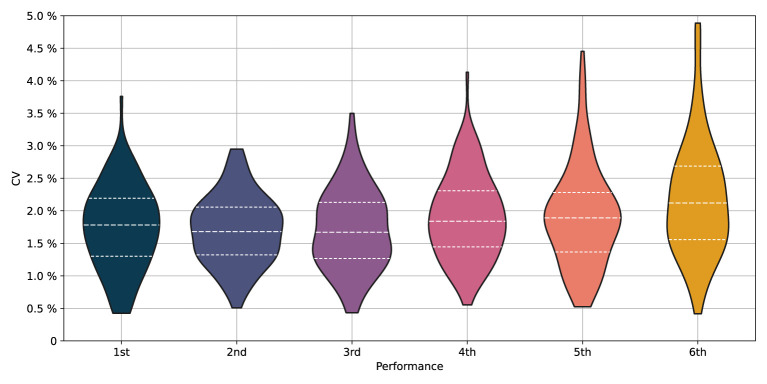
The distribution (violin plots; kernel density estimation) of coefficient of variation (CV) of the split times as function of performance, i.e., placement. The results include 636 boat crews from 106 A-finals. The bottom and top of the violins represent the minimum and maximum values of CV per placement. Dashed lines are used to indicate the lower quartile (Q1), median (Q2), and upper quartile (Q3).

Figure 2 illustrates the distribution, in terms of kernel density estimation, of CV per performance level. The distribution plots are cut at the maximum and minimum values of CV per placement. Dashed lines in Figure 2 indicate the lower quartile (Q1), median (Q2), and upper quartile (Q3). Q1, Q2, and Q3 of all medallists are smaller than the corresponding values of all non-podium finishers.

**Figure 3:**
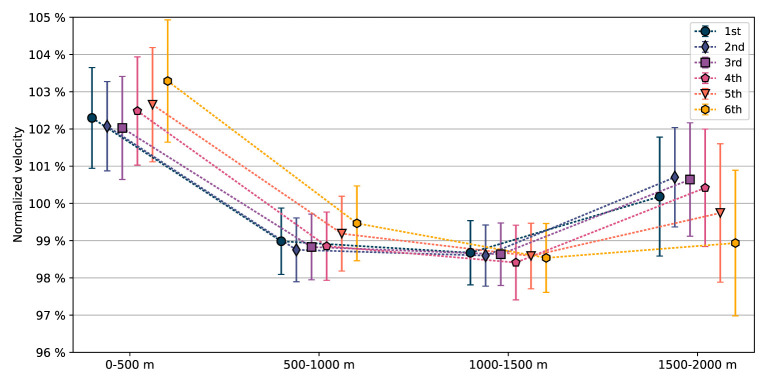
Mean normalized velocities, i.e., the mean velocity in a 500 m segment compared to the mean velocity of the full 2,000 m race distance, Equation (4), in the four segments of the race for all crews sorted on performance, 1st to 6th place (in 106 A-finals). Error bars indicate one standard deviation.

The mean normalized velocity profiles are presented as function of performance in Figure 3; numerical values are provided in Table 2. The medallists had, in general, lower normalized velocities in the first two segments of the race, and higher in the third and fourth segments when compared with the non-podium finishers. The differences in means between the medallists (1st, 2nd, 3rd place) and the non-podium finishers (4th, 5th, 6th place) yielded (*p*= 9 × 10^−9^, *d* = 0.46), (*p*= 4 × 10^−5^, *d* = 0.33), (*p* = 0.08, *d* = 0.14) and (*p*= 5 × 10^−9^, *d* = 0.47) in, respectively, the first, second, third, and fourth segments.

The higher normalized velocities in the first two segments yielded a larger mean positive split time, (*t*_3_+*t*_4_)−(*t*_1_+*t*_2_), among the non-podium finishers (4th: 2.57 s, 5th: 3.60 s, 6th: 5.46 s) compared to the medallists (1st: 2.39 s, 2nd: 1.56 s, 3rd: 1.59 s). Also, relative to each crew's mean 1,000 m time, $\frac{1}{2}\left(t_{1}+t_{2}+t_{3}+t_{4}\right)$, a difference between the non-podium finishers (4th: 1.23%, 5th: 1.73%, 6th: 2.61%) and the medallists (1st: 1.19%, 2nd: 0.75%, 3rd: 0.78%) was found. The difference in mean relative 1,000 m split time between the medallists (0.91%) and non-podium finishers (1.86%) yielded *p*= 3 × 10^−10^, *d* = 0.51.

In Table 3, CV for five subgroups—female and male crews (sex), singles, doubles/pairs and quads/fours (number of crew members)—are presented. The mean CV was somewhat smaller for female crews (1.81%) than for male crews (1.91%). This difference yielded *p* = 0.06, *d* = 0.15. Accordingly, the mean SRD was smaller for female crews (6.3%) than for male crews (6.7%), with *p* = 0.03, *d* = 0.18. The mean CV and SRD were larger for single crews (CV = 1.91%, SRD = 6.6%) compared to doubles/pairs (CV = 1.83%, SRD = 6.4%) and quads/fours (CV = 1.85%, SRD = 6.4%). However, comparisons of the CV of these groups all yielded *p*≥0.3, *d* ≤ 0.11; comparisons of the SRD all yielded *p*≥0.5, *d* ≤ 0.08.

**Table 3:** Mean CV (standard deviation), mean SRD (standard deviation), Equation (3), and the mean standard deviation, *t*_*std*_, of the four segment times, and mean normalized velocities in the four segments of the race, 0–500 m (*v*_1_), 500–1,000 m (*v*_2_), 1,000–1,500 m (*v*_3_), and 1,500–2,000 m (*v*_4_), for all boat crews, for female crews, for male crews, for singles, for doubles/pairs, and for quads/fours.

	**n**	**CV (%)**	**SRD (%)**	***t*_*std*_**	***v*_1_ (%)**	***v*_2_ (%)**	***v*_3_ (%)**	***v*_4_ (%)**
All crews	636	1.86 (0.71)	6.5 (2.4)	1.88 s	102.5	99.0	98.6	100.1
Female crews	312	1.81 (0.70)	6.3 (2.4)	1.92 s	102.4	99.0	98.6	100.1
Male crews	324	1.91 (0.71)	6.7 (2.5)	1.85 s	102.6	98.0	98.5	100.1
Singles	180	1.91 (0.75)	6.6 (2.6)	2.08 s	102.5	99.2	98.7	99.8
Doubles/pairs	270	1.83 (0.70)	6.4 (2.4)	1.86 s	102.4	98.9	98.6	100.1
Quads/fours	186	1.85 (0.67)	6.4 (2.3)	1.72 s	102.5	99.0	98.4	100.4

**Figure 4:**
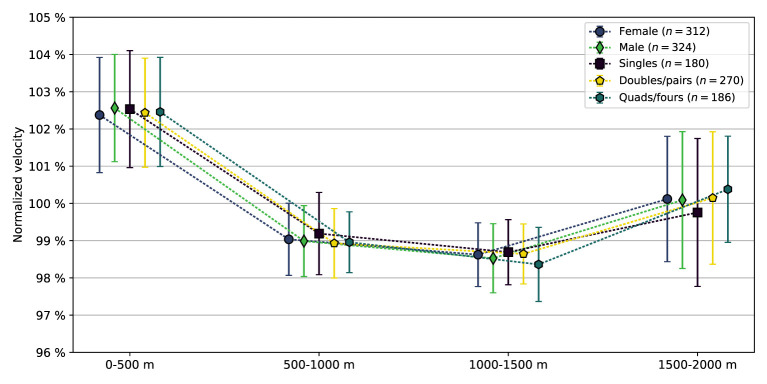
Mean normalized velocities, i.e., the mean velocity in a 500 m segment compared to the mean velocity of the full 2,000 m race distance, Equation (4), in the four segments of the race for all female and male boat crews, as well as the three groups of crew members (singles: 1 crew member, doubles/pairs: 2 crew members, quads/fours: 4 crew members). Error bars indicate one standard deviation.

In Figure 4, the mean normalized velocity profiles of the five subgroups are presented; numerical values are provided in Table 3. The differences in the normalized velocities between female and male boat crews were all minor (*p*≥0.1, *d* ≤ 0.13). Compared to doubles/pairs, singles had higher normalized velocity in the second segment (*p* = 0.01, *d* = 0.26) and lower normalized velocity in the fourth segment (*p* = 0.03, *d* = 0.21). Compared to quads/fours, singles had higher normalized velocity in the second (*p* = 0.02, *d* = 0.24) and third (*p*= 9 × 10^−4^, *d* = 0.35) segments, and lower normalized velocity in the fourth segment (*p*= 7 × 10^−4^, *d* = 0.36). Doubles/pairs had higher normalized velocity than quads/fours in the third segment (*p*= 0.001, *d* = 0.32).

**Figure 5:**
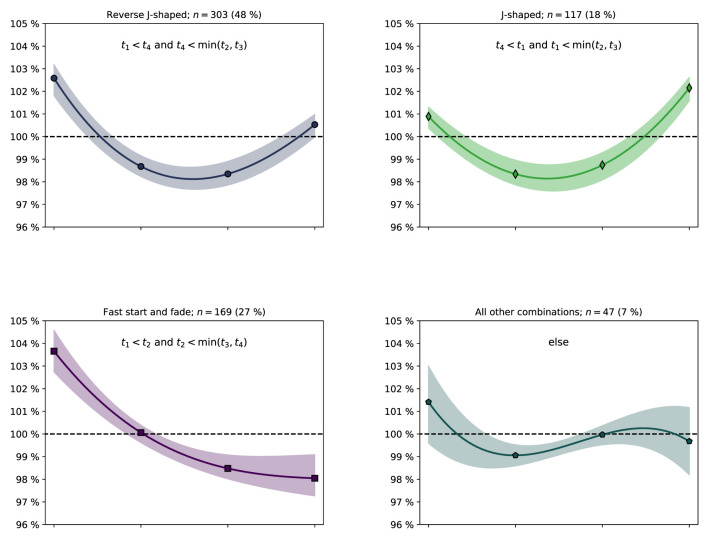
Illustrations of pacing profiles for the crews that fit into the reverse J-shaped **(upper left)**, standard J-shaped **(upper right)** and fast start and fade **(lower left)** pacing profile groups (all other combinations group; **lower right**), cf. Table 4. Markers: Mean normalized velocities in the four segments of the race. Solid line: Third order polynomial fit based on the four mean values. The shaded area is bordered by third order polynomial fits based on the lower (Q1) and upper (Q3) quartiles of the normalized velocities in the four segments of the race.

In Table 4, we categorize the velocity profiles in terms of three commonly found pacing profiles in rowing; the reverse J-shaped (fastest-slow-slow-fast), normal J-shaped (fast-slow-slow-fastest) and a positive pacing profile here denoted fast start and fade (fastest-fast-slow-slow). All but 47 crews fell into one of these three categories (93%). For 497 of 636 crews (78%), the first 500 m segment was the fastest of the four 500 m segments. Illustrations of the categorized pacing profiles, based on the normalized velocities (mean values and upper and lower quartiles) in each group, are presented in Figure 5.

**Table 4:** Pacing profile classification of the 636 considered boat crews.

**Abbreviations**	**Profile**	**Criterion**	**n**
r-J	Reverse J-shaped	*t*_1_<*t*_4_ and *t*_4_ < min(*t*_2_, *t*_3_)	303 (48%)
J	J-shaped	*t*_4_<*t*_1_ and *t*_1_ < min(*t*_2_, *t*_3_)	117 (18%)
FS	Fast start and fade	*t*_1_<*t*_2_ and *t*_2_ < min(*t*_3_, *t*_4_)	169 (27%)
else	All other combinations	different to the above	47 (7%)

*The pacing profile of a crew is classified based on the split times in the four segments of the race, t_1_ (0 to 500 m), t_2_ (500–1,000 m), t_3_ (1,000–1,500 m) and t_4_ (1,500–2,000 m)*.

Pacing profiles sorted on performance are presented in Table 5. The resulting *p*-values from chi-squared tests of independence are provided in Table 6. The difference in distribution of pacing profiles between some of the placements were significant, in particular for the last place which had a distribution that was different to all other placements (*p* ≤ 0.03). The difference in distribution of pacing profiles of all medallists combined [r-J: 163 (51%), J: 71 (22%), FS: 59 (19%), else: 25 (8%)] and the non-podium finishers combined [r-J: 140 (44%), J: 46 (14%), FS: 110 (35%), else: 22 (7%)] yielded *p*= 5 × 10^−5^.

**Table 5:** Pacing profile as function of final placement of the 636 considered boat crews.

**Abb**.	**1st**	**2nd**	**3rd**	**4th**	**5th**	**6th**
r-J	46 (43%)	63 (59%)	54 (51%)	54 (51%)	47 (44%)	39 (37%)
J	19 (18%)	26 (25%)	26 (25%)	19 (18%)	19 (18%)	8 (8%)
FS	32 (30%)	11 (10%)	16 (15%)	23 (22%)	37 (35%)	50 (47%)
else	9 (8%)	6 (6%)	10 (9%)	10 (9%)	3 (3%)	9 (8%)

*Profiles and criteria for the abbreviations are provided in Table 4*.

**Table 6:** *p*-values from chi-squared tests of independence comparing the distribution of the pacing profiles between the different final placements, cf. Table 5.

	**1st**	**2nd**	**3rd**	**4th**	**5th**	**6th**
1st	-	0.002	0.07	0.5	0.3	0.03
2nd	0.002	-	0.5	0.07	3 × 10^−4^	8 × 10^−9^
3rd	0.07	0.5	-	0.5	0.003	2 × 10^−6^
4th	0.5	0.07	0.5	-	0.06	7 × 10^−4^
5th	0.3	3 × 10^−4^	0.003	0.06	-	0.02
6th	0.03	8 × 10^−9^	2 × 10^−6^	7 × 10^−4^	0.02	-

The pacing profiles of the five subgroups presented in Figure 4 and Table 3 are provided in Table 7. Chi-square tests of independence yielded no significant difference in the pacing profile distributions between female and male crews (*p* = 0.5). The difference between singles and doubles/pairs was also insignificant (*p* = 0.2), as was the difference between doubles/pairs and quads/fours (*p* = 0.4). The difference in profiles between singles and quads/fours yielded *p* = 0.03.

**Table 7:** Pacing profiles of various subgroups.

**Abb**.	**Female**	**Male**	**1x**	**2x/2-**	**4x/4-**
r-J	140 (45%)	163 (50%)	76 (42%)	126 (47%)	101 (54%)
J	60 (19%)	57 (18%)	28 (16%)	55 (20%)	34 (18%)
FS	86 (28%)	83 (26%)	59 (33%)	71 (26%)	39 (21%)
else	26 (8%)	21 (6%)	17 (9%)	18 (7%)	12 (6%)

*Profiles and criteria for the abbreviations are provided in Table 4*.

### Segment Performance

**Figure 6:**
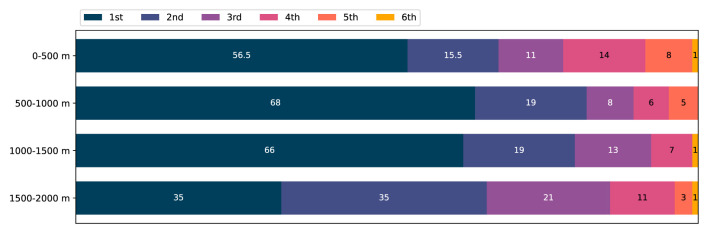
Segment winners sorted on race performance: the number of 1st, 2nd, 3rd, 4th, 5th, and 6th place finishers that are the fastest boat crew in each of the four 500*m* segments. 106 A-finals considered. Total number of segment wins: 1st place 225.5 (53%), 2nd place 88.5 (21%), 3rd place 53 (13%), 4th place 38 (9%), 5th place 16 (4%), 6th place 3 (1%).

In Figure 6, the fastest crews in each of the four segments of the race are presented in terms of the final placement. In 225.5 of the 424 considered segments (53%), the fastest crew was also the winner of the race^1^. The winner particularly dominated the two mid-race sections; in 68 of the considered finals (64%), the fastest boat from 500 m to 1,000 m was also the winner of the race, and in 66 finals (62%), the fastest boat from 1,000 m to 1,500 m was the winner.

**Figure 7:**
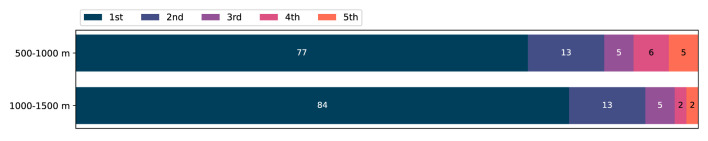
The number of race winners that are placed at the 1st, 2nd, 3rd, 4th, and 5th at 1,000 m and 1,500 m in the 106 considered A-finals. The position of the winning crew at 500 m is equal to the top bar of Figure 6 (at 2,000 m all winners are placed first).

Figure 7 is a supplement to Figure 6 and presents the position of the winners at 1,000 m and 1,500 m into the race. In 77 finals (73%), the leading crew at 1,000 m won the race, and in 84 finals (79%), the leading crew at 1,500 m won the race.

## Discussion

This study provides novel information on how rowers in A-finals of World and European championships apply their pacing strategy. 106 finals were considered, in which all had six competing boat crews. We used the coefficient of variation (CV) of the 500 m intermediate times to quantify the evenness of the pacing profiles. Analyses of an additional pacing variation parameter, the sum of the relative differences (SRD) of the 500 m split times, were overall consistent with analyses of the CV. We did not find significant differences in the CV of female vs. male crews—although females paced somewhat more evenly than men and had smaller SRD (*p* = 0.03, *d* = 0.18)—or in the CV of various crew members (singles vs. doubles/pairs vs. quads/fours), cf. Table 3. However, more even pacing profiles were evident for the medallists compared to that of the 4th–6th places, implying that the pacing pattern discriminates athletes at the highest performance level in World class rowing, cf. Figures 1, 2 and Table 2.

The mean pacing profile of all crews, in the subgroups (sex, crew members), and for all placements except the last, followed a reverse J-shape, cf. Figures 3, 4 and Tables 2, 3. In 78% of the crews, the first 500 m segment was the fastest of all four 500 m segments. These findings are in line with studies of previous rowing events (Garland, 2005; Brown et al., 2010; Muehlbauer et al., 2010; Muehlbauer and Melges, 2011). Moreover, segment analyses revealed the winner's dominance in the relatively slower mid-race sections, cf. Figure 6. Notably, for the winners, being the fastest crew in the last segment was considerably less common compared with the three other segments, and in 79% of the finals, the winner was also leading at the 1,500 m mark, cf. Figure 7. This is consistent with A-finals of previous World championships (2003–2007) and Olympic games (2004, 2008), studied by Brown et al. (2010), who found that 78% of winners were placed first at the 1,500 m mark. We note the lack of drafting benefits in rowing, which is an important difference between rowing and several other mass start sports, where the outcome often comes down to a so-called endspurt phenomenon, e.g., 1,500 m running (Casado et al., 2021). Consequently, there seems to be limited good reasons for saving energy for an endspurt (Tucker, 2009), a likely reason why the regular J-shaped pacing profile, often seen in other endurance sports of similar duration, does not seem to be that common in rowing, cf. Figure 5 and Table 4. Interestingly, in a recent rundown of the regattas at the 2021 Tokyo Olympics, Kleshnev (2021) showed that the mean pacing profile was J-shaped. Kleshnev (2021) also noted that the Tokyo Olympics was the second fastest of 28 World regattas since 1993, and the pacing profiles were characterized as “much more even” than previous events.

From a pure mechanical point of view, our main finding that performance depends on the evenness of the pacing profile, seems very likely. Considering the streamlined vessels and the mean velocities—which ranged from 4.1 m/ s to 6.0 m/ s in the considered events—the resistance force on the boat and crew, *F*, should be dominated by viscous forces that can be approximated as (Faltinsen, 2005),


(6) $$\begin{array}{l} F=\frac{1}{2}\rho CSu^{2}. \end{array}$$

Here ρ is the fluid density, *u* is the velocity, *C* is a dimensionless coefficient and *S* is a characteristic surface area, e.g., the friction forces along the wet hull are expressed with a friction coefficient, *C*_*f*_, and wet hull surface, *S*_*w*_. Alternatively, if pressure forces dominate—for instance the aerodynamic drag forces on the rower's upper body—the expression may be written in terms of a form drag coefficient, *C*_*d*_, and the projected frontal area, *A*. In either case, Equation (6) predicts that these viscous drag forces are proportional to velocity squared, *F*∝*u*^2^. Consequently, the power (*P*; the rate at which a force does work), needed to overcome the total resisting force in rowing, is approximately proportional to velocity cubed, *P*∝*u*^3^. More refined models and experimental investigations support these assumptions, e.g., *P*∝*u*^3.2^ (di Prampero et al., 1971), *P*∝*u*^2.95^ (di Prampero, 1986), *P*∝*u*^2.8^ (Affeld et al., 1988), *P*∝*u*^2.7^ (Hofmijster et al., 2007), and *P*∝*u*^2.92^ (Hill and Fahrig, 2009). Arguments have been made that the exponent is somewhat smaller if a model of actual rowing is considered (Shephard, 1998; Hill and Fahrig, 2009). Nevertheless, a highly non-linear relation between power and velocity is obtained. Consequently, for a given mean power output, the mean velocity is maximized when rowing at a constant velocity, i.e., constant power (even pacing strategy), assuming similar conditions over the course of the race. This is impossible for two obvious reasons; 1) initially, the boat must be accelerated from rest; 2) intracyclic velocity variations are inevitable consequences of the propulsive mechanisms of rowing. However, these effects may be treated separately from changes to the mean stroke velocity during the greatest part of the race. Typically, the initial acceleration phase of a 2,000 m regatta is limited to the very start of the race, increasing the time spent on the first 50 m of the race by 1–4 s compared to the mean 50 m split time, but not affecting later 50 m split times (Thompson, 2014, Figure 11.1). Moreover, the intracyclic velocity variations may be treated as representing an additional resistance component—which requires an additional power output—compared to traveling the boat constantly at the mean stroke velocity (Hofmijster et al., 2018), with an associated increase in the net mechanical power of 2–10% (Nigg, 1984; Sanderson and Martindale, 1986; Hofmijster et al., 2007; Hill and Fahrig, 2009; de Brouwer et al., 2013).

A reason why relatively fast starts (despite the initial acceleration phase) are common in rowing, may be due to the fact that rowing regattas are lane-based mass start events with both mental and hydrodynamic arguments for leading, not following. Since the crews are faced backwards, being upstream of competitors is a visual advantage. Therefore, a lead may give a sense of control with positive mental feedback which could influence performance (Schiphof-Godart et al., 2018). Clear favorites that are likely winners in many race scenarios may enjoy the confidence in taking an early lead to have visual control of the race. This may explain why the winners had slightly larger CV, relatively faster starts and more often applied the fast-start and fade pacing strategy, compared to the other medallists, cf. Figures 1–3, and Tables 2, 5, 6. In addition to mental aspects, there are also hydrodynamic arguments for uneven pacing. Incident waves from the competitors' boats may yield both a larger resistance force and make it more challenging to row effectively for a crew downstream. The risk of incident waves from competing boats may be estimated by considering the Kelvin angle, that is, the angle between the boundary of the wave system and course of a vessel (Faltinsen, 2005). Assuming deep water conditions, the Kelvin angle is $\arcsin\frac{1}{3}\approx$ 19°. If the lane width is 12.5 m and the boats are placed in the center of their lanes, the course direction distance from the wave propagation of the upstream boat to the stern of the downstream boat, must be less than 35 m to avoid incident waves from the upstream boat on the downstream boat. This distance is reduced if the upstream crew position the boat closer to the line of buoys. Consequently, if competitors apply a very fast start, incident waves is a risk worth noting if applying a more conservative pacing strategy. However, in general, the smallest risk of incident waves from competing boats throughout the race is achieved by going as fast as possible for the whole race distance, not just the start.

## Conclusion

Medal winners in major rowing championships pace with smaller variation than their competitors. This may imply that a more even pacing profile, than what is typically applied by non-podium finishers, is advantageous in rowing.

## Data Availability Statement

The dataset supporting the conclusions of this article will be made available by the corresponding author, without undue reservation.

## Ethics Statement

Ethical review and approval or written informed consent were not required for the study on human participants in accordance with the local legislation and institutional requirements.

## Author Contributions

FM collected and analyzed the data. Both authors contributed to the design of the study, wrote the manuscript, and approved the submitted version.

## Conflict of Interest

The authors declare that the research was conducted in the absence of any commercial or financial relationships that could be construed as a potential conflict of interest.

## Publisher's Note

All claims expressed in this article are solely those of the authors and do not necessarily represent those of their affiliated organizations, or those of the publisher, the editors and the reviewers. Any product that may be evaluated in this article, or claim that may be made by its manufacturer, is not guaranteed or endorsed by the publisher.

## Footnotes

^1^In one event (W19 W4x), both the first and second place had the equal fastest time at the 500 m mark.
